# Convergent evolution of dynamic camouflage: humidity-responsive shell colouration in arboreal snails

**DOI:** 10.1186/s40851-026-00266-7

**Published:** 2026-06-25

**Authors:** Taro Yoshimura, Takenori Sasaki

**Affiliations:** 1 The University Museum, The University of Tokyo, 7-3-1 Hongo, Bunkyo-ku, Tokyo, 113-0033 Japan; 2 Graduate School of Science, The University of Tokyo, 7-3-1 Hongo, Bunkyo-ku, Tokyo, 113-0033 Japan; 3 Faculty of Science and Technology, Keio University, 3-14-1 Hiyoshi, Kohoku-ku, Yokohama City, Kanagawa Prefecture 223-8522 Japan

**Keywords:** Dynamic camouflage, Hygrochromic colour change, Periostracum, Arboreal snail, Convergent evolution, Refractive index matching, Phenotypic plasticity

## Abstract

Terrestrial organisms employ diverse camouflage strategies, yet the fluctuating humidity and light conditions of arboreal habitats demand dynamic adaptations. This study investigated a novel mechanism of dynamic camouflage in the arboreal snails *Hypselostyla camelopardalis* (Camaenidae) and *Reinia variegata* (Clausiliidae). These phylogenetically distant species exhibit a reversible hygrochromic change: their mottled shell patterns disappear upon wetting, turning uniform dark brown, and rapidly reappear as they dry. Using a multimodal approach—including confocal laser microscopy, scanning electron microscopy, and spectrophotometry, this study shows that the colour change is associated with structural modifications within the bilayered organic periostracum. In the white regions, hydration fills microscale voids and smooths surface irregularities, effectively matching the refractive index of the periostracum and increasing light transmittance. While camouflage in terrestrial gastropods was previously considered static, our findings reveal an environmentally responsive system that dynamically adjusts to ambient moisture. This mechanism parallels strategies observed in certain insects, and is consistent with functional convergent evolution. Furthermore, the water-responsive thin-film structure of the snail shell provides a biological blueprint for the development of bioinspired smart materials, such as humidity-sensitive coatings and adaptive optical technologies.

## Introduction

Organisms have evolved a wide range of defensive strategies to evade predation, among which camouflage is one of the most widespread adaptations [1–5]. Camouflage encompasses several distinct strategies, including crypsis, where an organism blends into its background [6–8]; countershading, which reduces three-dimensionality by masking body shadows [9–11]; and mimicry, where an organism imitates the visual features of its surroundings [6, 12, 13]. Most camouflage strategies are static, meaning that once established, their visual characteristics remain unchanged unless the organism moves [14]. However, some animals have evolved dynamic camouflage, allowing them to alter their coloration or patterns in response to environmental changes. Well-documented examples include pigment cell expansion in chameleons [15], chromatophore regulation in cephalopods [16], and pigment adjustments in intertidal crabs [17].

The colouration of molluscan shells arises from multiple mechanisms, including pigment absorption, fluorescence, translucency, scattering, diffraction, and structural refraction [18, 19], with a detailed survey of these factors provided by Tilley [20]. Since the Cambrian explosion and the emergence of image-forming eyes, predatory selection has exerted strong pressure on the coloration and patterns of molluscan shells, favouring both camouflage and aposematic signalling [21, 22]. The effectiveness of shell coloration and patterning as camouflage against visual predators such as crabs, fish, and birds has been widely demonstrated [23–34]. Among molluscs, one of the most common camouflage strategy is background-matching crypsis [35–38]. Additionally, shell coloration in the near-ultraviolet range may be subject to selective pressure from predators capable of UV perception [39].

**Fig. 1:**
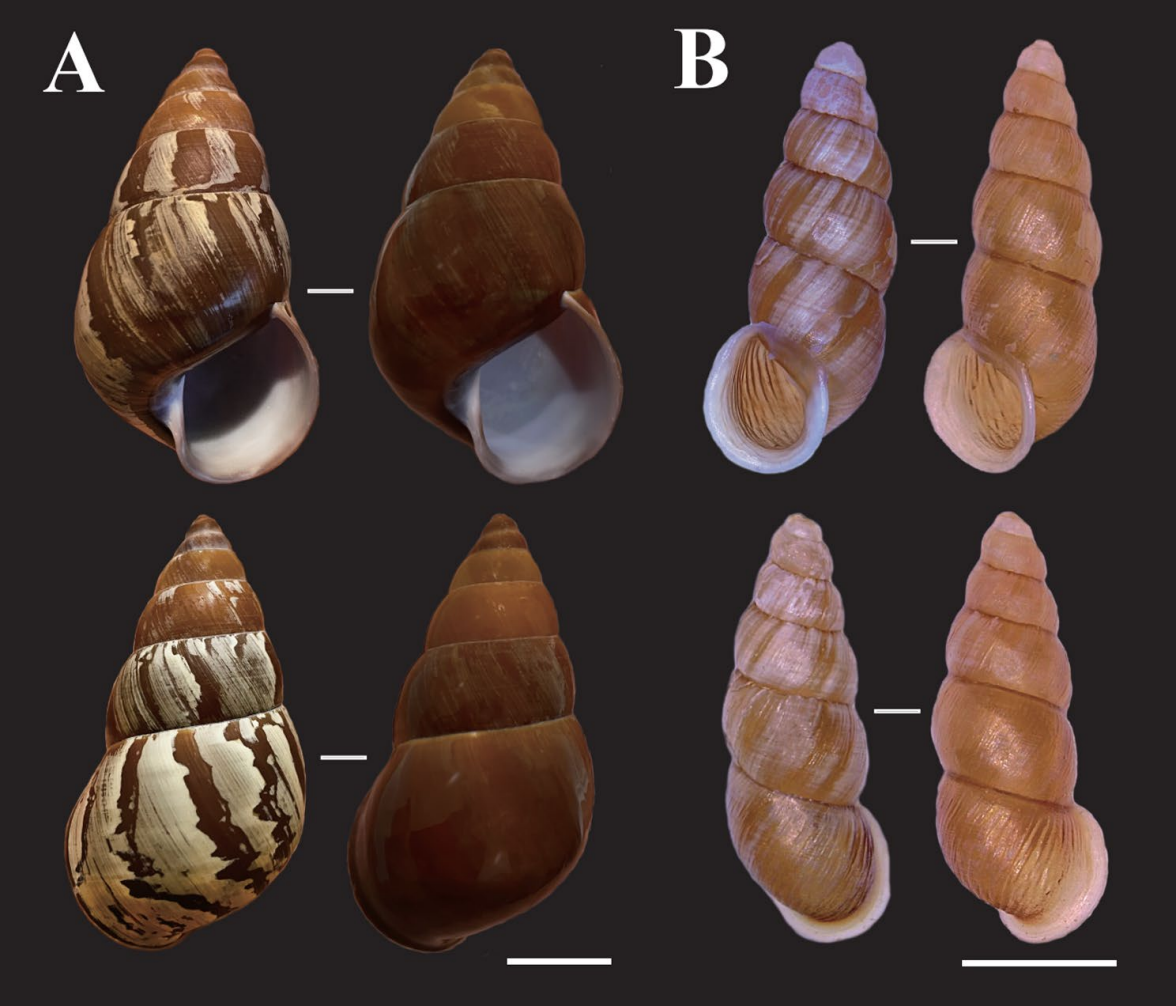
Changing pattern by water-absorbing in arboreal snail shells. **(A)** *Hypselostyla camelopardalis* (Broderip, 1841) (Helicoidea: Camaenidae), **(B)** *Reinia variegata* (Adams, 1868) (Clausilioidea: Clausiliidae). Left and right are dry and wet condition, respectively. Scale bar = 10 mm

Arboreal snails exhibit moisture-dependent behaviour, becoming active during rainfall and resting on bark or leaves when dry [40]. In most terrestrial gastropods, shell coloration is considered static, as the pigments are typically embedded within the dense calcified layers or a solid, non-porous organic matrix [18, 41]. Even in species with pale or translucent shells, the appearance generally remains unchanged regardless of environmental moisture because their periostracum lacks the specialized microstructure required for dynamic optical shifts [42]. Among these taxa, the phylogenetically distant species, *Hypselostyla camelopardalis* (Helicoidea: Camaenidae) and *Reinia variegata* (Clausilioidea: Clausiliidae), display a distinctive hygrochromic colour change, where their mottled white and brown shells turn uniformly dark brown upon wetting, with the original pattern reappearing as they dry (Fig. 1). While this phenomenon is scarcely reported in molluscs, similar reversible colour changes occur in insects. In *Dynastes hercules* (Scarabaeoidea: Scarabaeidae), hydration alters the refractive index of a porous, light-scattering structure, darkening the elytra [43–46]. Cassidine chrysomelids, including *Aspidomorpha tecta*, *Charidotella egregia*, *Deloyala*, and *Metriona*, shift from gold to red upon dehydration due to the collapse of broadband cuticular reflectors [47–49]. Crowson [50] noted that this phenomenon is widespread among cassidine chrysomelids. From an adaptive perspective, several studies have suggested that such moisture-dependent colour changes serve as dynamic camouflage to match fluctuating backgrounds. In *D. hercules*, it has been proposed that the blackening of the elytra under high humidity allows the beetles to match the darkening of wet bark, thereby maintaining crypsis during rainfall [44, 45]. This hypothesis is further supported by reports that their activity increases in humid conditions, similar to the behaviour seen in arboreal snails. In contrast, the underlying mechanisms and adaptive significance of hygrochromic colour change in arboreal snails remain largely unexplored.

This study specifically focuses on how structural changes in the periostracum, the outermost organic layer of the shell, influence its optical properties. The periostracum serves as an interface between the shell and the external environment, primarily composed of proteins and chitin [51]. During shell formation, it acts as a substrate for crystal deposition, making it an intrinsic feature of all molluscan shells [52]. In certain taxa adapted to low pH, low temperatures, or high pressures, the periostracum is notably thickened to prevent carbonate dissolution [42, 53, 54]. Given the diverse habitats occupied by molluscs, including deep-sea, freshwater, and terrestrial environments, the periostracum likely serves a broad range of adaptive functions [55].

To elucidate the mechanisms of this colour change, we employ three complementary approaches. First, scanning electron microscopy (SEM) is used to examine the surface and cross-sectional microstructure of the periostracum, identifying structural features associated with the phenomenon. Second, confocal laser microscopy enables real-time visualization of structural changes, capturing the dynamics of different shell layers under varying humidity conditions. Third, spectrophotometric analyses quantify changes in light transmission induced by moisture variations. Collectively, these methods provide insight into the physical basis of shell colour change.

This study advances our understanding of dynamic camouflage mechanisms in terrestrial molluscs, offering new perspectives on adaptive strategies in response to environmental variability. Furthermore, the humidity-responsive colour change mechanism identified here may have biomimetic applications, potentially informing the development of tunable optical coatings [56, 57] and self-healing camouflage materials [58, 59]. More broadly, our findings contribute to the understanding of organismal adaptation to humidity fluctuations, shedding light on functional convergent evolution while also informing the design of bioadaptive materials.

## Methods

### Materials

The Hypselostyla camelopardalis specimens examined in this study are historical museum specimens curated at The University Museum, The University of Tokyo (UMUT). These shells were acquired and accessioned into the museum’s permanent collection before 29 December 1993, the date on which the Convention on Biological Diversity entered into force. As these materials are classified as pre-CBD specimens, they are not subject to the current access and benefit-sharing (ABS) requirements under the Nagoya Protocol or the Philippine Wildlife Resources Conservation and Protection Act (Republic Act No. 9147). No new biological materials were collected from the wild for this study, and all research was conducted using these long-established institutional archives.

Specimens of *Reinia variegata* (Adams, 1868) (Clausilioidea: Clausiliidae) were collected alive snails from Ehime Prefecture, Japan, in June 2021, in accordance with local regulations. *H. camelopardalis* was primarily utilized for detailed experiments due to its larger shell size, which facilitated more precise operational procedures. To mitigate potential degradation of the periostracum, all shell specimens were stored for approximately one year under controlled environmental conditions (20 °C, 50–60% relative humidity). Importantly, the shells were not exposed to ethanol or other organic solvents during processing or storage to preserve the integrity of the periostracal microstructures. Voucher specimens were registered at UMUT under the following registration numbers: RM34576 (*H. camelopardalis*) and RM34577 (*R. variegata*).

### Definition of hydration conditions

To ensure quantitative reproducibility, the hydration states used in this study were strictly defined as follows. The dry condition refers to specimens equilibrated at a controlled room temperature of 20 °C ± 1 °C and relative humidity (RH) of 50–60% for at least 24 h prior to measurement. Under these conditions, the shell surface was completely dry, and the characteristic mottled white pattern was fully visible. The wet condition is defined as the state of saturation achieved by immersing the shell specimens in distilled water at 20 °C for 30 s. Measurements in the wet condition were conducted immediately after removing the specimens from water and gently blotting excess surface droplets, ensuring the periostracum remained saturated.

### Scanning electron microscopy

Scanning electron microscopy (SEM) was used to observe the periostracal microstructure. Samples were coated with osmium (HPC-20 Plasca CVD equipment, Vacuum Device) for FE-SEM observation (GeminiSEM 500, Carl Zeiss). The FE-SEM instrument was equipped with an Oxford Instruments XMax detector with an accelerating voltage of 15 kV and working distance of 7 mm in low-vacuum mode (N2, 40 Pa). A VPSE detector was used at magnifications from 200× to 2500×.

### Laser confocal microscopy

To investigate fine topographical changes of the periostracum surface under dry and wet conditions, images were acquired using a Keyence 3D laser-scanning confocal microscope (VK-X1000 series). The samples were observed without conductive coating to monitor the reversible morphological variations corresponding to hydration states.

### Spectrophotometry

To quantify the light transmittance of the periostracum, spectral measurements were performed under both dry and wet conditions. Shell specimens were decalcified in 2% EDTA for 48 h (*H. camelopardalis*) following the method of Ruthensteiner [60] to isolate the organic periostracum. The isolated membranes were rinsed, and fragments corresponding to the white and pigmented regions were dissected. These fragments were mounted between two quartz slides to maintain flatness and ensure UV transparency.

Transmittance spectra (300–800 nm) were obtained using a double-beam UV-VIS spectrophotometer (UV-1700, Shimadzu, Japan). Due to the small shell size and fragile nature of the isolated membranes in *R. variegata*, which prevented stable mounting, spectrophotometric analysis was performed exclusively on *H. camelopardalis* (*n* = 4 individuals). For each individual, measurements were taken at five distinct points for each of the four experimental conditions: dry white, dry pigmented, hydrated white, and hydrated pigmented regions. This resulted in a total of 20 measurements per condition to ensure representative data.

## Results

### Surface microstructure

**Fig. 2:**
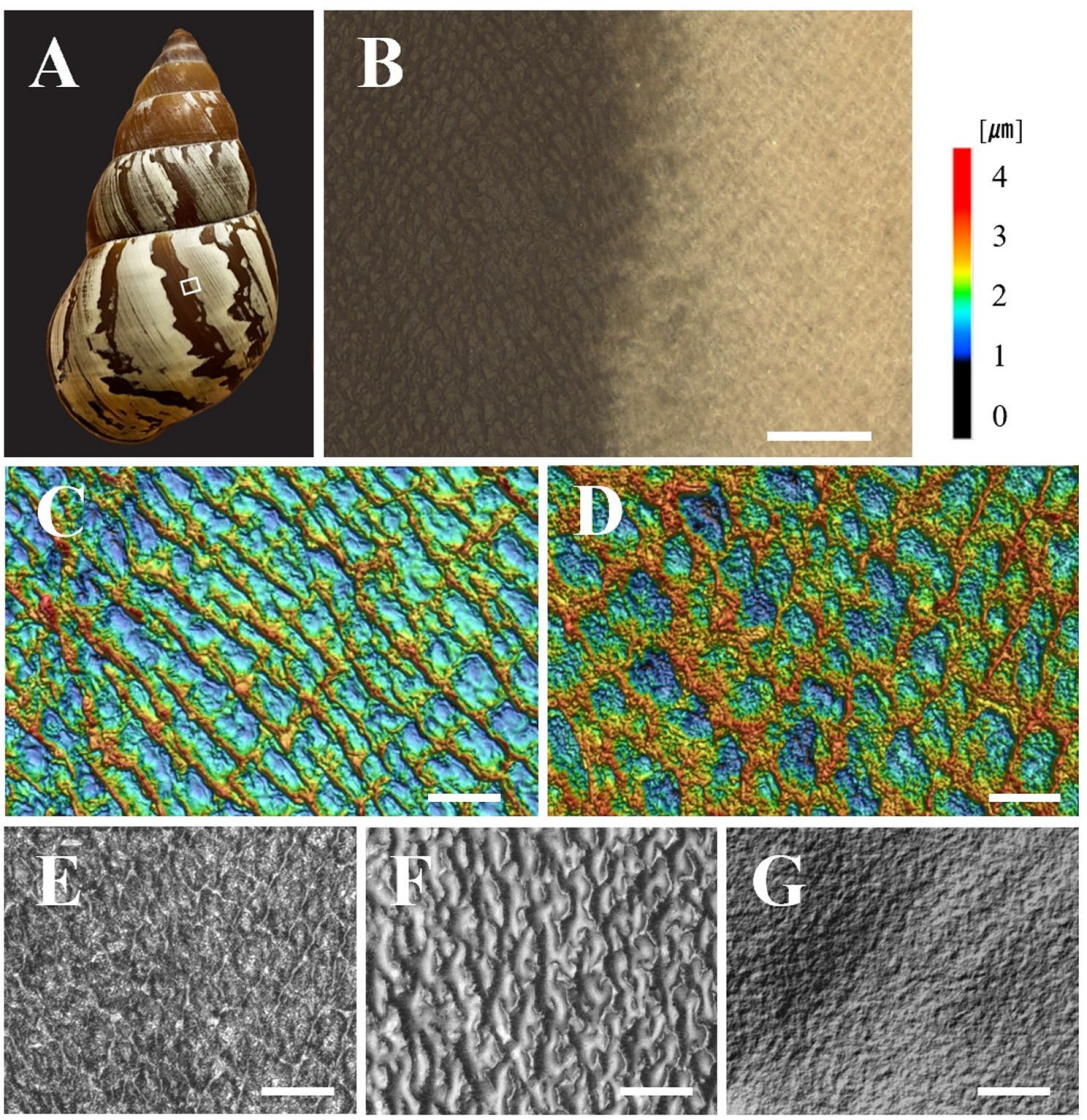
Surface microstructure of the periostracum in *Hypselostyla camelopardalis* observed using a laser confocal microscope.** (A)** A dry specimen of *H. camelopardalis* and the region of observation. **(B)** Boundary between the pigmented and white regions of the shell. **C**,** D.** Fine surface structures and topography of the periostracum. **(C)** Pigmented region. **(D)** White region. **E-G.** Changes in the surface structure of the periostracum in the white region during hydration. **(E)** Dry condition. **F**,** G.** After approximately 3 and 6 s of hydration, respectively. The color scale in **C** and **D** indicates surface height variations. Scale bar: **B** = 300 μm, **C**,** D** = 20 μm, **E-G** = 30 μm

**Fig. 3:**
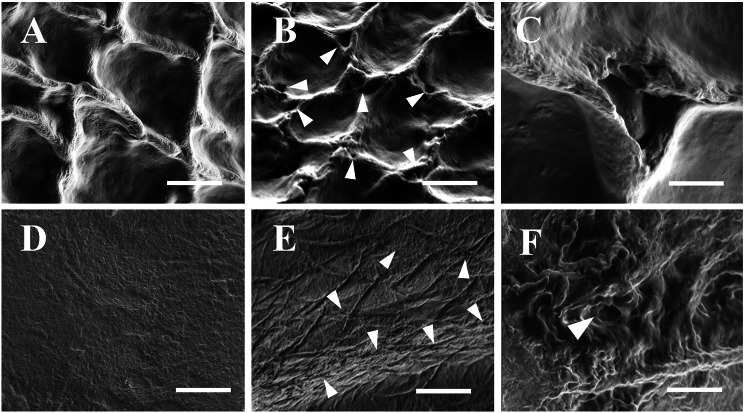
Distribution of micropores on the periostracum surface observed using FE-SEM.** A-C.** *Hypselostyla camelopardalis*. **(A)** Pigmented region. **(B)** White region. **(C)** Magnified view of micropores. **D-F.** *Reinia variegata*. **(D)** Pigmented region. **(E)** White region. **(F)** Magnified view of micropores. Arrows indicate micropores. Scale bar: **A**,** D** = 5 μm, **B**,** E** = 8 μm, **C** = 2 μm, **F** = 1 μm

Confocal laser microscopy and FE-SEM observations revealed fine wrinkles on the periostracum surface of *Hypselostyla camelopardalis* and *Reinia variegata*. In *H. camelopardalis*, a grid-like pattern of wrinkles at approximately 10 μm intervals was observed (Fig. 2A-D). Confocal laser measurements indicated that the height variation reached approximately 2 μm in the transparent regions (Fig. 2C; here, transparent region refers to the part of the periostracum through which pigments deposited in the calcium carbonate shell layer are externally visible) and up to approximately 4 μm in the white regions (Fig. 2D). In contrast, the periostracum of *R. variegata* exhibited almost no wrinkles in the transparent regions (Fig. 3D), while irregular wrinkles of approximately 1–4 μm were observed in the white regions (Fig. 3E).

To examine changes in surface morphology during hydration, we tracked the absorption process in the periostracum of *H. camelopardalis* using confocal laser microscopy. In the dry condition, a grid-like wrinkle pattern was present (Fig. 3E). Within ~ 3 s of hydration, these wrinkles connected and transitioned into broader, more regularly aligned folds (Fig. 3F), with a surface height variation of approximately 1–2 μm. After an additional 3 s of hydration, the large folds disappeared, and surface-height variation decreased to less than 1% of that in the initial dry condition (Fig. 2G).

FE-SEM observations revealed the presence of microscale pores on the white regions of the periostracum in both species. In *H. camelopardalis*, pores with a maximum diameter of 200–700 nm were sparsely distributed, with approximately 3–5 per 10 μm² (Fig. 3A-C). In *R. variegata*, smaller pores (approximately 40–100 nm in diameter) were more densely packed, with 70–80 per 10 μm² (Fig. 3D-F).

### Cross-sectional structure

**Fig. 4:**
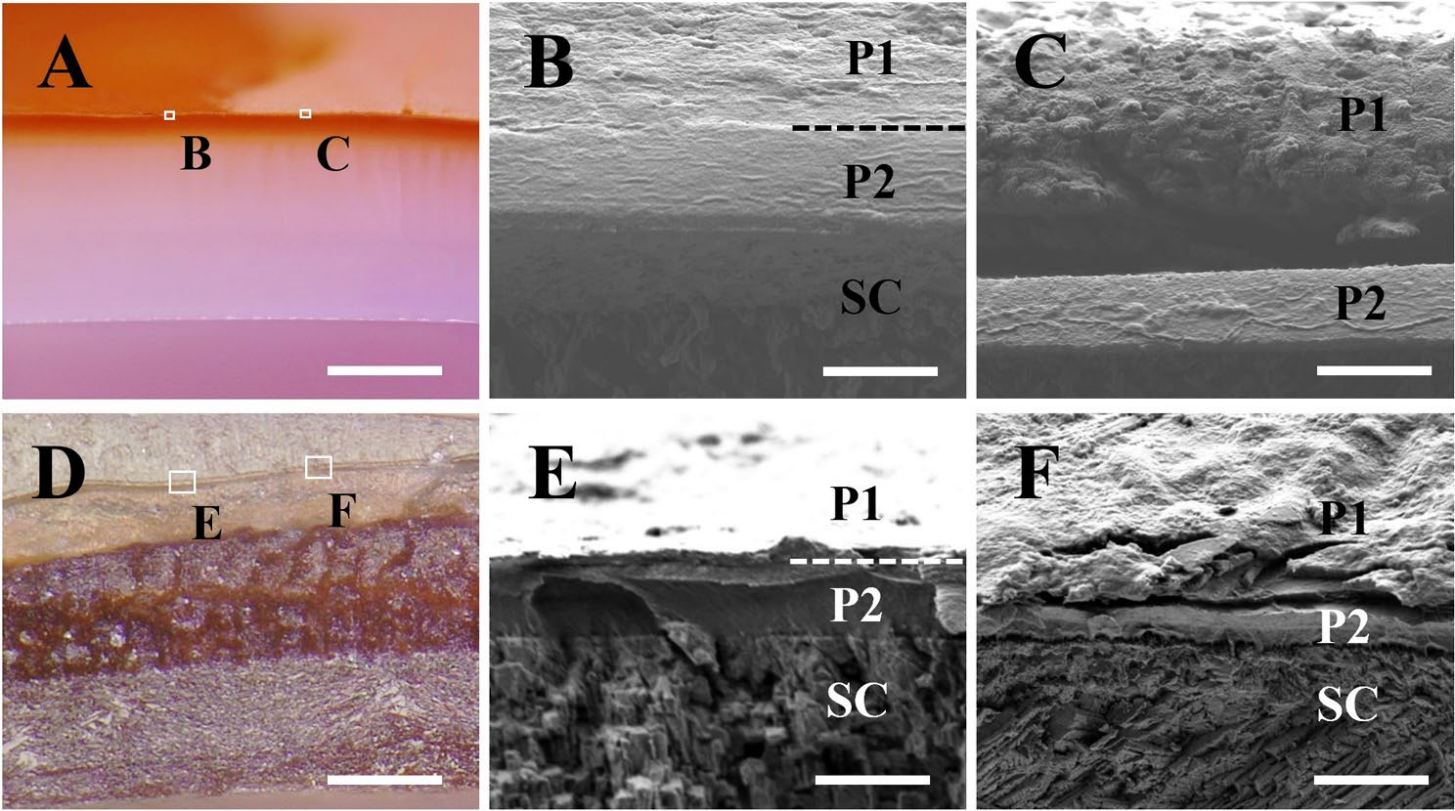
Cross-sectional observations of the shell using a digital microscope and FE-SEM. ** A-C.** *Hypselostyla camelopardalis*. **(A)** Digital microscope image of the shell. The outer crystalline layer exhibits a uniformly distributed light brown pigment. **(B)** SEM image of the periostracum in the pigmented region. The boundary between the two layers of the periostracum is indicated by a dashed line. **(C)** SEM image of the periostracum in the white region. **D-F.** *Reinia variegata*. **(D)** Digital microscope image of the shell. The crystalline layer is distinguishable into three layers, with uniform pigment distribution within each layer. **(E)** SEM image of the periostracum in the pigmented region. The boundary between the two layers of the periostracum is indicated by a dashed line. **(F)** SEM image of the periostracum in the white region. P1: Outer periostracal layer, P2: Inner periostracal layer, SC: Shell crystalline layer. Scale bar: **A** = 1 mm, **B**,** C** = 20 μm, **D** = 400 μm, **E**,** F** = 10 μm

Digital microscopy of shell cross-sections showed that the brownish pigmentation in both *H. camelopardalis* and *R. variegata* was not associated with the periostracum but rather deposited within the underlying crystalline layer (Fig. 4A, D). The periostracum itself consisted of white and transparent regions, while pigments were uniformly embedded in the underlying crystals (Fig. 4A, D).

FE-SEM analysis of cross-sections revealed a bilayered periostracal structure in both species. In the white regions, (1) the outer periostracal layer (Fig. 4P1) exhibited a sponge-like porous structure (Fig. 4C, F), and (2) an irregular gap was present between the outer and inner periostracal layers (Fig. 4C, F). In contrast, the brown regions showed a dense, compact periostracal structure with no visible pores, and no gaps between the outer and inner layers (Fig. 4B, E). Additionally, in both species, the inner periostracal layer was tightly adhered to the underlying crystalline shell layer (Fig. 4).

### Changes in transmittance due to hydration

**Fig. 5:**
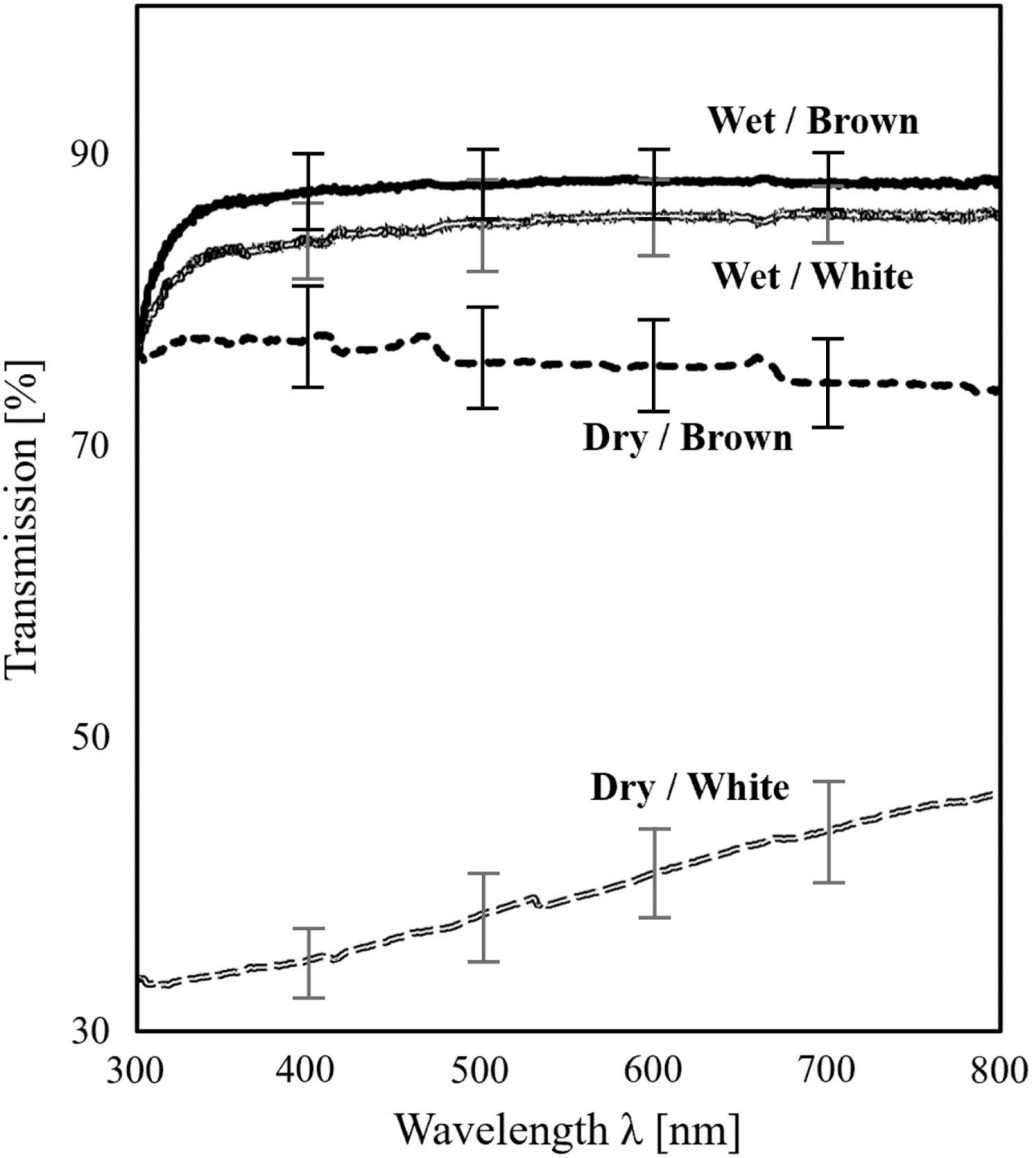
Light transmittance of the periostracum in *Hypselostyla camelopardalis* under different hydration conditions. The graph displays the mean transmittance across the 300–800 nm range. Error bars represent the standard deviation (± SD) calculated from 20 measurements across four specimens, plotted at 400, 500, 600 and 700 nm. The four lines represent: Wet/Brown (hydrated pigmented region; solid line), Wet/White (hydrated white region; double line), Dry/Brown (dry pigmented region; dotted line), and Dry/White (dry white region; double-dotted line). Note that ‘Brown’ indicates the regions where the underlying shell pigments are visible through the periostracum due to its high transmittance

Light transmittance in the ultraviolet to visible range (300–800 nm) markedly increased upon hydration in *H. camelopardalis* (Fig. 5). The mean transmittance values across the measured range were as follows: 37.4% for dry white regions, 73.9% for dry pigmented regions, 85.3% for hydrated white regions, and 87.8% for hydrated pigmented regions.

In the dry condition, a substantial difference in mean transmittance (36.5%) was observed between the white and pigmented regions. In contrast, upon hydration, the periostracum became uniformly translucent, and the difference in transmittance between the two regions diminished to a negligible level. This optical shift was particularly pronounced in the ultraviolet range, where the hydrated periostracum allowed nearly maximal transmission regardless of the underlying shell pigmentation.

## Discussion

### Mechanism of humidity-responsive camouflage

**Fig. 6:**
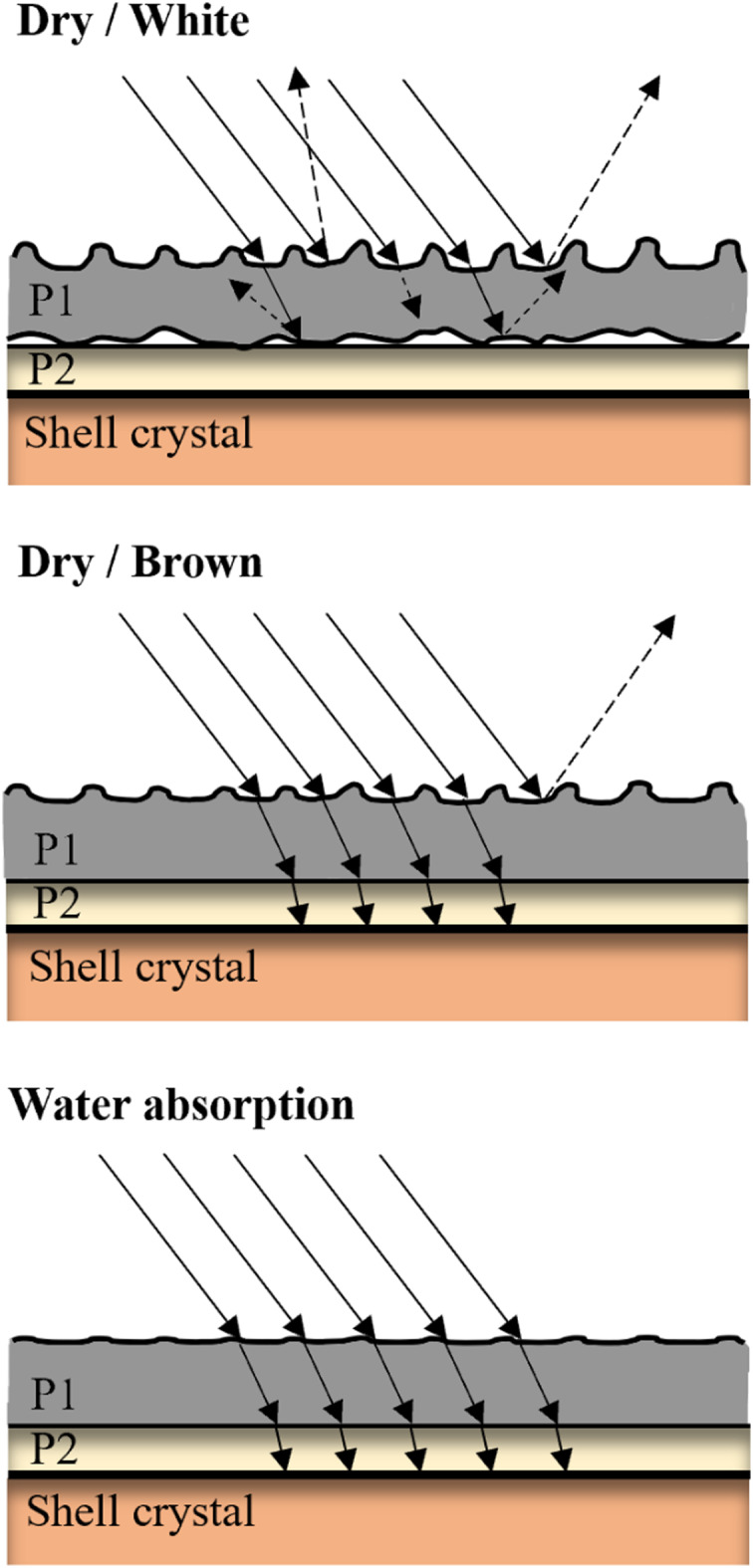
Proposed mechanism of color change in the periostracum due to hydration. In the dry condition, the periostracum in the white region exhibits: (1) Unevenly spaced gaps between the outer (P1) and inner (P2) periostracal layers, (2) A highly irregular surface topography, and (3) A low-density outer layer (**P1**), causing light scattering that obscures the pigment deposited in the underlying shell crystal layer. In contrast, both the pigmented periostracum in the dry condition and the hydrated periostracum exhibit: (1’) A tightly packed structure with no gaps between the outer (**P1**) and inner (**P2**) periostracal layers, (2’) A smoother surface topography, and (3’) A denser outer layer (**P1**), allowing light transmission and making the underlying shell crystal pigments visible

This study indicates that the humidity-responsive camouflage observed in arboreal snails is attributable to structural changes in the shell periostracum. Specifically, alterations in (a) surface morphology, (b) outer layer density, and (c) the air layer at the boundary between the outer and inner layers were observed. First, surface observations using scanning electron microscopy (SEM) and confocal laser microscopy revealed that, under dry conditions, the white regions of the periostracum develop fine voids and wrinkled surface irregularities (Fig. 3). In contrast, under humid conditions, these voids become filled with water, and the wrinkles smooth out (Fig. 2E-G). Next, the microstructure of the outer layer was found to be spongy and rich in low-density air pockets in white regions (Fig. 4), which likely contribute to light scattering. Furthermore, an air gap of approximately 5–8 μm was identified between the outer and inner layers in the white regions (Fig. 4C, F). Spectrophotometric measurements confirmed that light transmittance in the white regions substantially increases in humid conditions (Fig. 5). This increased transparency reduces visual contrast between the white pattern elements and the underlying pigmented shell layer, producing a more uniformly dark appearance that may better match wet substrates (Fig. 6).

This humidity-responsive camouflage is achieved through the bilayered structure of the periostracum and its moisture-responsive properties. The outer layer contains microscale pores that facilitate water absorption (Fig. 3C, F), forming a porous network that dynamically modulates optical properties through refractive index matching. When water (*n* ≈ 1.33) replaces air (*n* = 1.0) within the pores, the refractive index mismatch with the periostracum (*n* ≈ 1.5) is reduced, thereby suppressing light scattering and enhancing transparency. Although this study primarily quantified these properties through transmittance measurements, these results are physically coupled with the surface reflectance that defines the shell’s visual appearance. According to the principle of energy conservation in a non-absorbing medium, a decrease in light scattering necessarily results in increased transmittance and a concomitant decrease in backscattered reflectance. Therefore, the high transmittance observed in the hydrated state (Fig. 5) directly corresponds to a reduction in reflectance, explaining the visual transition from a bright, scattering white to a dark, pigment-dominated appearance. This optical coupling suggests that transmittance data can serve as a useful proxy for evaluating hygrochromic reflectance shifts relevant to dynamic camouflage. Meanwhile, the denser inner layer (Fig. 4B, E) may regulate moisture retention and release, ensuring the reversibility of the colour change. This structure enables rapid adaptations to fluctuations in ambient humidity and is likely to function as an effective camouflage strategy against visual predators [33, 34, 37].

The mechanism proposed in this study holds potential for applications in novel material development. By incorporating the microscale porous structure and layered configuration observed here, bioinspired materials with high hydrophilicity and reversible swelling-shrinking properties could be designed to alter transparency and reflectivity in response to humidity. For instance, mimicking the spongy structure of the outer layer could lead to the development of smart glass with adjustable light transmission [61] or adaptive camouflage materials [62]. Additionally, integrating the moisture-retaining function of the periostracum could facilitate applications in food packaging [63], wound dressings [64], or humidity-sensitive sensor materials [65]. Materials that dynamically modulate optical properties in response to humidity without external energy input may prove useful as next-generation smart materials with broad industrial potential.

### Convergent evolution of hygrochromic organic membranes

The two species of arboreal snails examined in the present study demonstrate that humidity-responsive camouflage has independently evolved across distant lineages. Arboreal environments are characterized by drastic fluctuations in humidity, which alter the visual properties of the substrate; for instance, tree bark significantly decreases in reflectance and becomes darker when wet. The hygrochromic change observed in these snail shells—shifting from mottled white to dark brown—closely tracks this environmental transition. This suggests a mechanism of dynamic background matching, wherein the snail minimizes its visual contrast against the substrate to evade detection [66, 67].

Arboreal snails face intense predation from visual hunters, particularly birds, which utilize sophisticated colour vision to detect prey against complex backgrounds [68, 69]. The role of shell coloration in bird predation has been extensively documented in common terrestrial snails such as *Cepaea nemoralis*, where shell morph frequencies shift in response to background colour to reduce conspicuousness [70, 71]. In contrast to such species, the majority of terrestrial gastropods possess a significantly thinner periostracum that often undergoes progressive abrasion or complete delamination during the adult stage [42, 72]. Consequently, these species lack the persistent organic substrate necessary to harbor the micro-porous structures that facilitate refractive index matching upon hydration. The development of a thick, structurally complex, and wear-resistant periostracum in *H. camelopardalis* and *R. variegata* is thus a specialized adaptation, likely driven by the unique selective pressures of arboreal habitats where background coloration shifts dramatically with humidity. While *Cepaea* relies on static polymorphism, the hygrochromic snails in our study exhibit rapid phenotypic plasticity, allowing for near-instantaneous adaptation to short-term weather fluctuations.

The adaptive significance of such background matching is well-illustrated by the classic case of industrial melanism in the peppered moth, Biston betularia. In this species, selection favours phenotypes that match the reflectance of bark to avoid bird predation [73, 74]. While *Biston betularia* represents a genetic adaptation to long-term environmental shifts, hygrochromic snails exhibit phenotypic plasticity for an immediate response. Given that primary predators rely heavily on visual cues [75, 76], this mechanism of crypsis likely evolved under strong selective pressure, as seen in other molluscs where shell coloration is linked to predation pressure [33, 34, 77].

This form of physical camouflage is not unique to snails but is observed across diverse biological groups, including insects and amphibians. For example, the cuticle of certain beetles contains microscale structures that alter their refractive index upon moisture absorption [45, 78]. In scaled springtails, metallic lustre facilitates background assimilation under varying humidity [79]. Similarly, the keratinous microstructures in the skin of arboreal frogs modify light scattering in response to moisture [80, 81]. Furthermore, in certain spider species, humidity-induced changes in reflection reduce visibility under specific environmental conditions [82]. The occurrence of such humidity-dependent mechanisms across multiple taxonomic groups suggests that these are cases of functional convergent evolution rather than shared ancestry. Future comparative analyses of microstructures across these taxa, combined with molecular studies, will provide a more comprehensive understanding of the shared principles of humidity-responsive camouflage.

## Conclusions

This study identifies a previously unrecognised mechanism of dynamic hygrochromic camouflage in arboreal snail shells, driven by reversible structural modifications of the periostracum. This purely physical process relies on refractive index matching within the porous periostracum to rapidly modulate light scattering and transmission in response to hydration. Such a system allows these snails to maintain crypsis against fluctuating backgrounds without metabolic energy expenditure, potentially providing an important survival advantage against visual predators in arboreal environments. This environmentally responsive mechanism in terrestrial gastropods represents a remarkable instance of functional convergent evolution, paralleling similar moisture-dependent strategies observed in insects and other taxa. Beyond its ecological significance, the water-responsive properties of the snail shell may provide a blueprint for the development of passive smart materials, including humidity-sensitive optical coatings and adaptive biomimetic technologies. Ultimately, these findings fundamentally advance our understanding of flexible camouflage strategies and highlight the sophisticated ways in which organisms adapt to rapidly changing environmental conditions.

## Data Availability

The dataset supporting this research is openly available in the Dryad Digital Repository at 10.5061/dryad.gqnk98t10. For the purpose of peer review, the dataset supporting this manuscript is available at the following secure URL: http://datadryad.org/share/R1hMw3_FZEm_tQm-NNJC8q9An2HAf3qdr1Jj4xtrBX8.
